# Predicting surgical outcomes in spring assisted cranioplasty via finite element analysis and animal experiments

**DOI:** 10.1038/s41598-025-27092-9

**Published:** 2025-12-03

**Authors:** Wenjie Cheng, Bozhi Hou, Yuehua Li, Raymond Chung Wen Wong, Xiaojun Tang

**Affiliations:** 1 Department of Plastic Surgery, The Affiliated People’s Hospital of Ningbo University, 251, Baizhang Road, Ningbo, Zhejiang, 315040 China; 2 Faculty of Dentistry, National University of Singapore, 11 Lower Kent Ridge Road, Singapore, 119083 Singapore; 3 Department of Craniomaxillofacial surgery, Plastic Surgery Hospital, Chinese Academy of Medical sciences, Peking Union Medical College, No.33 Ba-Da-Chu Road, Beijing, 100043 China

**Keywords:** Sagittal craniosynostosis, Spring-assisted cranioplasty, Finite element analysis, Bone regeneration, Regression analysis, Biophysics, Computational biophysics, Preclinical research

## Abstract

Sagittal craniosynostosis, a congenital cranial suture disorder, is treated with spring-assisted cranioplasty (SAC), but optimal surgical parameters remain unclear. This study explores computational models to predict surgical outcomes of SAC by linking biomechanics with bone regeneration and cranial remodeling. Fifteen 3-week-old Sprague–Dawley (SD) rats underwent SAC with nickel-titanium springs (0–100 g forces). Bone regeneration was tracked via fluorescence labeling, while micro-CT scans measured cephalic index (CI), bone mineral density (BMD), and bone volume fraction (BV/TV). Finite element analysis (FEA) simulated stress and strain distributions. Regression models were established to predict the relationship between mechanical indicators and surgical outcomes. The 50 g group achieved optimal bone regeneration and cranial correction, while > 80 g forces risked bone damage. FEA indicates stress and strain over the bone are influenced by spring force, rat geometry, and bone density. Regression analysis revealed linear strain-CI relationships and quadratic strain-BV/TV and strain-BMD relationship. Moderate spring force (50 g) or strain (1.0%) enhances osteogenesis without structural compromise. Computational models provide a biomechanically grounded framework for SAC optimization, advancing precision treatment for sagittal craniosynostosis. Future studies should validate findings in disease-specific models and assess long-term outcomes.

**Supplementary Information:**

The online version contains supplementary material available at 10.1038/s41598-025-27092-9.

## Introduction

Sagittal craniosynostosis, the most prevalent form of craniosynostosis, constitutes 50–60% of nonsyndromic cases, with an incidence of approximately 1 in 2000–2500 live births^1,2^. Characterized by premature sagittal suture fusion, it results in an elongated, narrow skull (scaphocephaly)^3^, potentially leading to elevated intracranial pressure and neurodevelopmental impairments if untreated^4^. Traditional surgical interventions, though effective, are invasive, with significant trauma and recurrence risks. Since its introduction by Lauritzen et al. in 1998, spring-assisted cranioplasty (SAC) has emerged as a minimally invasive alternative, utilizing calibrated springs post-suturectomy to gradually expand the cranial vault. Clinical series (> 100 cases) demonstrate SAC’s efficacy in normalizing cephalic index (CI), with reduced operative time, blood loss, and recovery duration compared to full calvarial reconstruction^5^. Despite these advances, optimal SAC parameters—spring force, placement, and their impact on bone regeneration and cranial remodeling—remain poorly understood, posing a key research challenge.

Finite element analysis (FEA), a cornerstone of biomechanical modeling, simulates stress and strain in complex structures^6,7^. While widely applied in orthopedics to optimize implants and predict outcomes^8^, its use in SACs is limited. Jenson Jacob employed FEA to model cranial deformation under varying spring configurations and estimate CI changes^1^, yet gaps persist: many studies lack experimental validation and overlook individual bone density and cranial shape variations, hindering personalized simulations. Stress and strain, which are critical to bone regeneration and damage, are often neglected in this field^9^. This study addresses these deficiencies by integrating FEA with rat model experiments to analyze how spring force, cranial shape, incision site, and bone density influence stress and strain.

Regression analysis has been widely applied in the study of sagittal craniosynostosis to describe the relationships between surgical variables and outcomes, enabling precise prediction^10^. Integrating regression analysis with FEA can significantly increase both the accuracy and efficiency of predictions, as FEA can gather biomechanical data that can be validated by experiments, whereas regression analysis reduces computational costs^11^. By combining regression analysis with computational biomechanics, Jacob et al.predicted changes in human cranial CI in response to spring force^1^; Joel et al.investigated the relationship between biomechanics and bone regeneration^12^; and Levadnyi et al.explored the relationship between mechanical loading and bone damage^13^. These studies demonstrate that using computational models to establish a relationship between biomechanics and surgical outcomes can be feasible in spring-assisted cranioplasty.

This study aims to establish a multidimensional efficacy assessment framework (incorporating laboratory analyses and three-dimensional digital measurements) and develop FEA-based regression models for predicting the surgical outcomes of spring-assisted cranioplasty.

## Results

### General observations in animal experiment

All groups exhibited similar body weight trajectories throughout the study, with no significant intergroup differences in weekly measurements (one-way ANOVA, *p* > 0.05). Postoperative monitoring confirmed successful wound healing at cranial suture sites across all groups. Clinical evaluation revealed complete epithelialization within 7 days, with no signs of infection (e.g., erythema, purulent discharge) or device displacement.

**Fig. 1:**
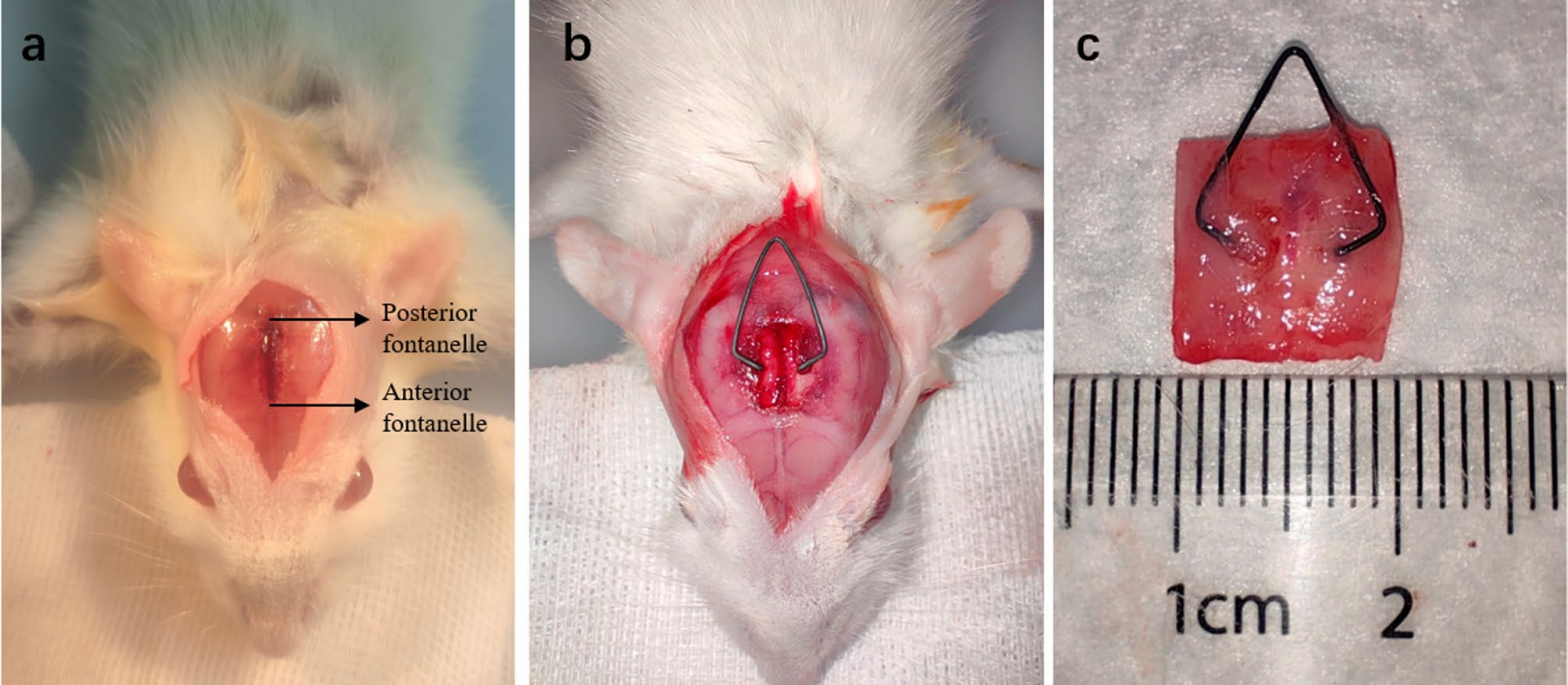
Surgical procedure. (**a**) Surgical approach incision and localization of the sagittal suture resection. (**b**) Placement of the spring implant. (**c**) Macroscopic view of the cranial sagittal suture region specimen.

At the 28-day endpoint, cranial specimens containing the sagittal suture were harvested. Each specimen measured approximately 1 × 1 cm and included the sagittal suture and adjacent cranial regions (Fig. 1).

### Bone fluorescence labeling

Fluorescence staining scans revealed distinct labeling patterns in the bone marrow and cortical bone regions at the sagittal suture defect margins. Clear fluorescent marker lines were observed at each time point, displaying green (tetracycline labeling), yellow (overlap region of tetracycline and calcein double labeling), and red (calcein labeling) bands. The yellow fluorescent bands, representing the overlap between calcein (injected on day 0) and tetracycline (injected on day 4), indicated new bone formation along the suture edges toward the defect gap during both the fluorochrome activation period and between injection time points. Notably, the amount of new bone formation from day 4 to day 14 was significantly greater than that from day 0 to day 4.

**Fig. 2:**
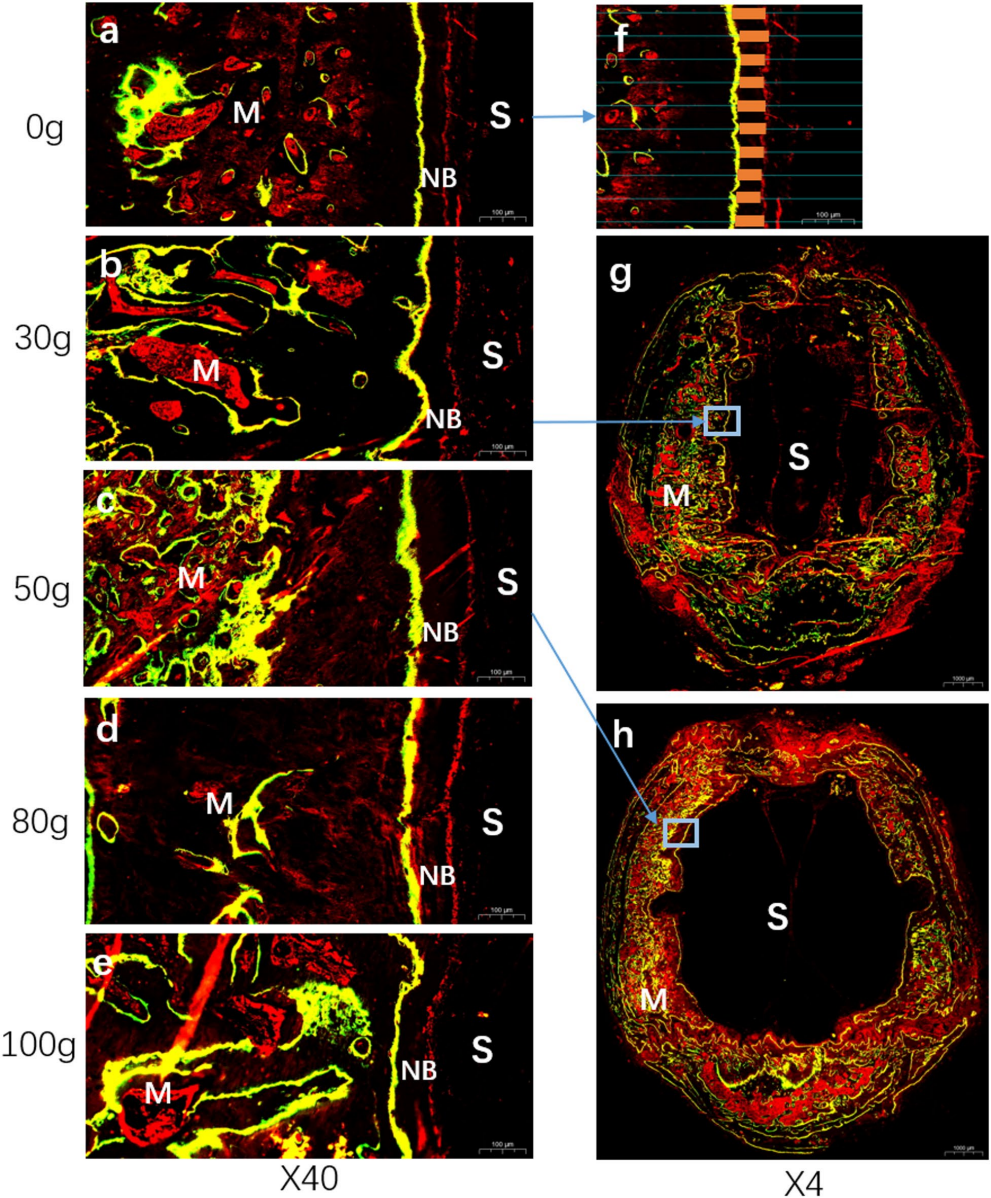
Fluorescent labeling analysis. (**a**)–(**e**) Fluorescence staining results on one side of the sagittal suture defect area under different spring forces. The scale bar in the lower right corner represents 100 μm. (**f**) Schematic diagram showing the measurement of the distance between the red fluorescence marker line and the yellow fluorescence marker line under 0 g spring force. (**g**) and (**h**) Staining results of the complete sagittal suture specimens under 30 g and 50 g spring forces. The scale bar in the lower right corner represents 1000 μm.

Under varying spring tension conditions, differences in bone marrow staining intensity (reflecting bone marrow stem cell activity) were observed, demonstrating that mechanical tension gradients significantly modulate osteoblast activity (Fig. 2). Using *ImageJ* software (Version 1.54f., National Institutes of Health, USA; https://imagej.nih.gov/ij/), the spacing between fluorescent bands was measured, revealing that the yellow/red band spacing (representing new bone growth from day 4 to day 14) in the 50 g group was significantly greater than that in other groups (Fig. 4a). One-way ANOVA confirmed statistically significant intergroup differences (*P* < 0.05). The 0 g group exhibited the smallest fluorescent band spacing, indicating minimal suture growth under tension-free conditions, while the 50 g group demonstrated the most significant suture expansion.

### 3D reconstruction analysis

**Fig. 3:**
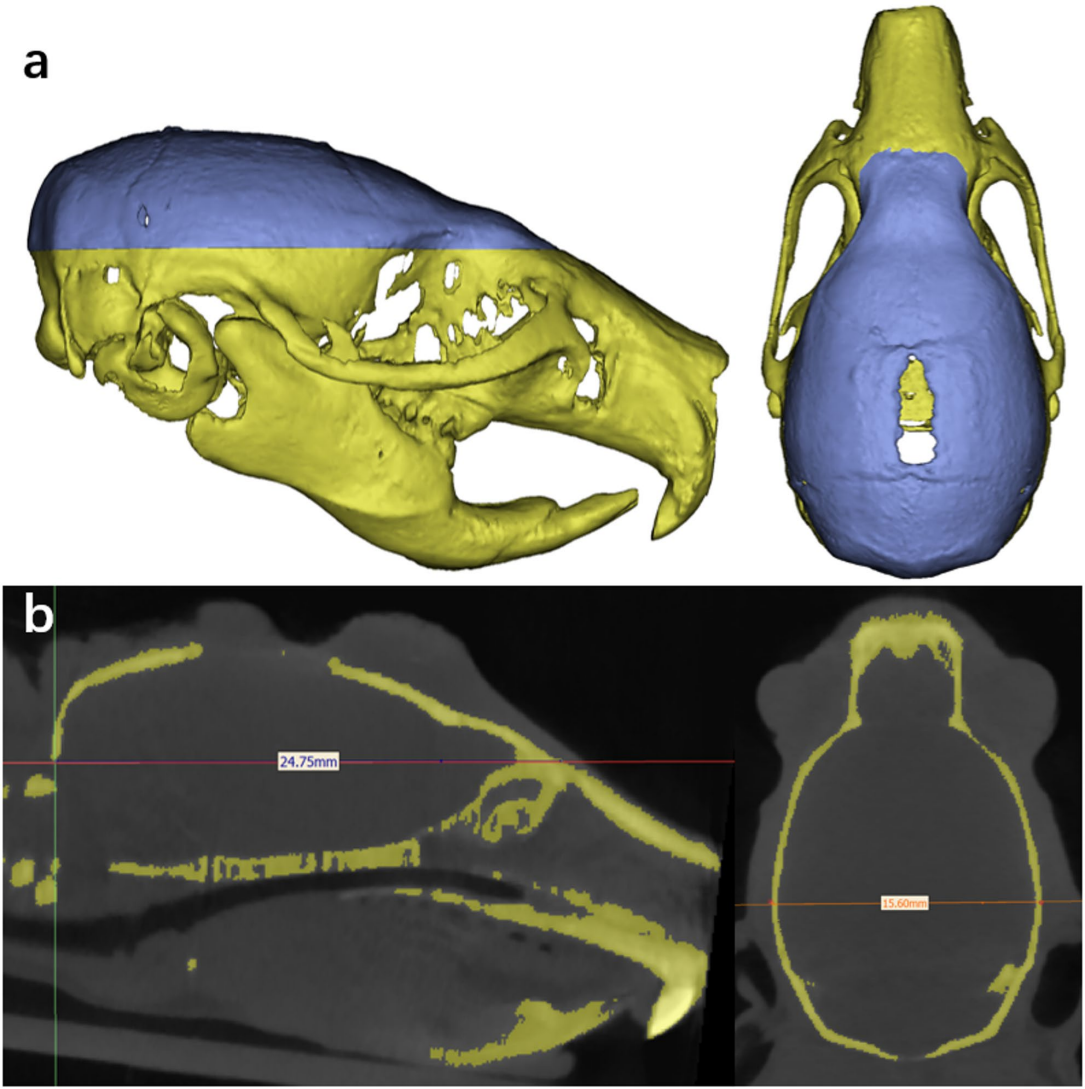
Three-dimensional reconstruction of the SD rat skull model. (**a**) Three-dimensional reconstruction image of the rat skull. The purple region represents the ROI (Region of Interest). (**b**) CT scan results. The yellow region indicates the cranial bone.

As shown in Fig. 3, the reconstructed cranial models clearly preserved the cortical outlines, enabling detailed morphological assessment. High-resolution renderings revealed that defect margins and newly formed bone were sharply defined, with no visible artifacts that could obscure boundary interpretation, ensuring the accuracy and precision in the postprocessing operation such as bone regeneration analyses and FEA.

**Fig. 4:**
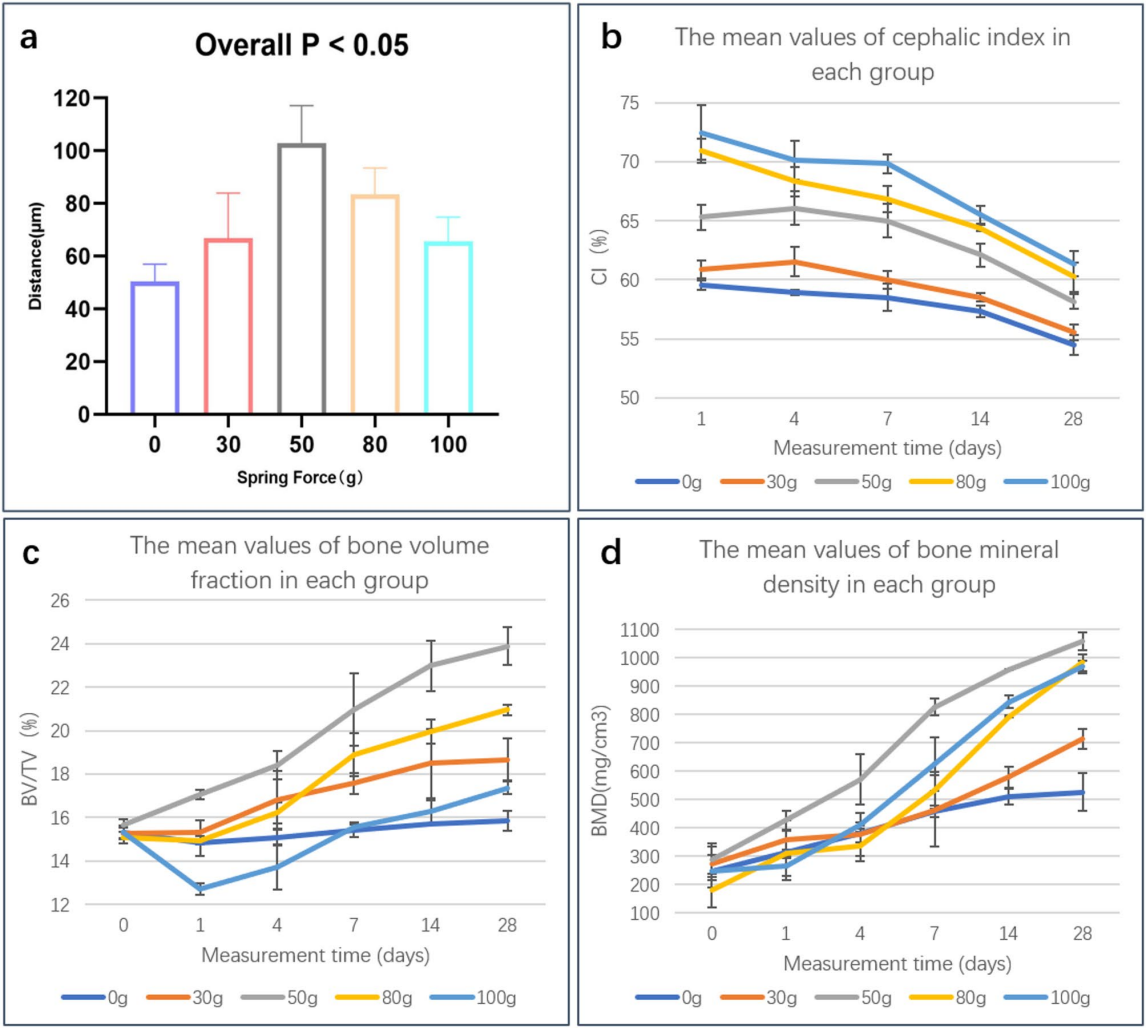
Surgical outcomes analysis. (**a**) Average fluorescence band spacing from day 4 to day 14 after suture expansion under different spring forces. (**b**) Mean cephalic index (CI) over time under different spring forces. (**c**) Mean bone volume fraction (BV/TV) over time under different spring forces. (**d**) Mean bone mineral density (BMD) over time under different spring forces.

As shown in Fig. 4, bone volume fraction (BV/TV) and bone mineral density (BMD) increased over time in all spring force groups, with the most pronounced and rapid improvements observed in the 50 g group. Rats subjected to identical spring forces exhibited comparable BV/TV and BMD trends. Cephalic index (CI) data demonstrated that higher spring forces correlated with greater CI values at each time point, followed by a more pronounced decline over time.

### Finite element analysis

**Fig. 5:**
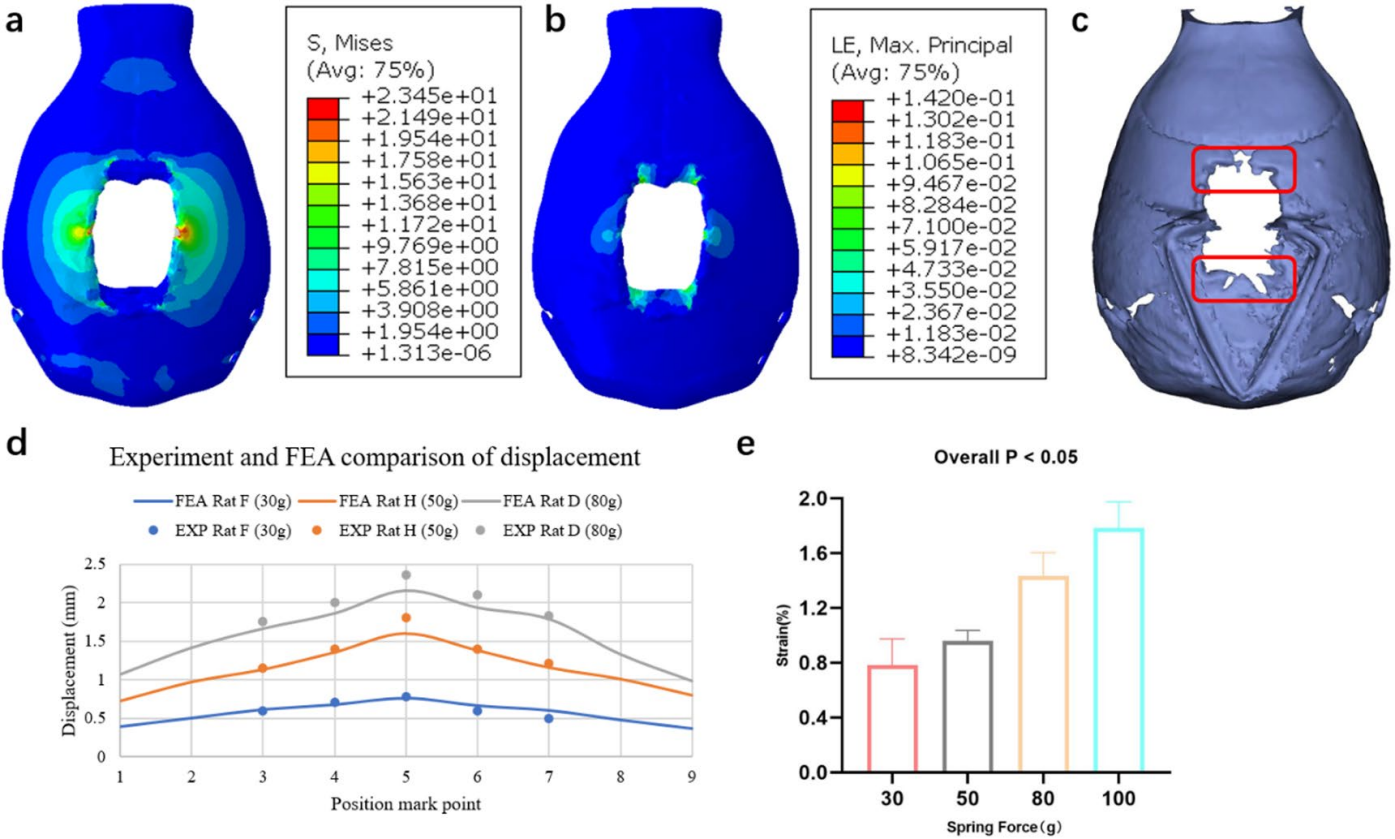
Finite element analysis results. (**a**) Distribution and magnitude of Von Mises stress. (**b**) Distribution and magnitude of maximum principal strain. (**c**) Experimental 3D reconstruction. Regions in red bounds present bone crack. (**d**) Validation of FEA results by comparing with experimental results. (**e**) Variation of mechanical strains in different spring forces and rats. Note that panels (**a**) and (**b**) represent different physical quantities (von Mises stress and maximum principal strain). The corresponding color scales are therefore not directly comparable and are shown only to illustrate the spatial distribution patterns.

The Von Mises stress distribution for a rat with a spring force of 50 g is shown in Fig. 5a. The highest stress (23.45 MPa) is observed near the spring, and some regions exceed the yield stress of approximately 12 MPa^13^. To further investigate areas with significant deformation, the logarithmic strain distribution is presented in Fig. 5b. The maximum value of the principal strain (LE) exceeds 0.3, indicating a large strain^14^ and suggesting that bone fractures likely occurred in high-strain regions. Experimental 3D reconstruction confirmed the presence of cracking in the spring contact area and at the four corners of the wound, as shown in Fig. 5c.

A comparison between the FEA predictions and experimental observations is shown in Fig. 5d. At lower spring forces, the simulation results align well with the experimental data. However, at higher spring forces, the wound deformation exceeds the simulation predictions, suggesting localized brittle fracture. Despite these discrepancies, the overall error between the simulation and experimental results remains within 10%.

The correlation between the average principal strain and spring force is significant; however, strain fluctuations within an approximately 15% range are observed under identical spring forces due to differences in rat variation, such as wound shape, cranial morphology and bone density, as illustrated in Fig. 5e.

### Regression analysis

The scores of the regression models for the relationships between biomechanics and surgical outcomes are summarized in Table 1. Spring-based and strain-based regression model are compared in day 4 and day 28. Scores in red mark are selected to be the best model to demonstrate the relationship. These findings demonstrate that surgical outcomes are effectively predicted by strain and spring force in both short-term and long-term cases. The regression scores for the strain model were higher than those for the spring force model with improvements ranging from 0.01 to 0.09.

**Table 1:** Regression analysis scores. Spring-based and strain-based regression model are compared in day 4 and day 28. Scores in bold are selected to be the best model to demonstrate the relationship.

		Spring force			Strain		
		Linear	Quadratic	Exponential	Linear	Quadratic	Exponential
DAY 4	CI	**0.89**	0.90	0.85	**0.94**	0.94	0.91
	BV/TV	0.12	**0.75**	0.19	0.14	**0.84**	0.22
	BMD	0.39	0.39	0.65	0.46	0.49	**0.76**
DAY 28	CI	**0.87**	0.88	0.87	**0.89**	0.91	0.87
	BV/TV	0.51	**0.75**	0.56	0.51	**0.76**	0.59
	BMD	0.68	**0.74**	0.71	**0.74**	0.78	0.75

Statistical analysis was further applied to the long-term relationships between strain and surgical outcomes, as shown in Table 2. Coefficient of constant term, first-order term (× 1) and second-order term (× 2) presented. The result is considered statistically significant except for a large *p* value (greater than 0.10) for the constant term of the strain-BV/TV relationship. However, the *p* values of first- and second-order terms are still sufficient to demonstrate a quadratic relationship between strain and BV/TV.

**Table 2:** Statistics analysis of strain-based regression model in day 28. Coefficient of constant term, first-order term (x1) and second-order term (x2) presented.

		Coeff	Std err	t	P >|t|	[0.025	0.975]
CI	const	1.97	0.55	3.56	< 0.01	0.78	3.17
	× 1	6.65	0.47	14.06	< 0.01	5.63	7.68
BV/TV	const	0.90	0.67	1.43	0.177	− 0.47	2.27
	× 1	6.90	1.43	4.82	< 0.01	3.78	10.01
	× 2	− 2.54	0.75	− 3.40	< 0.01	− 4.17	− 0.91
BMD	const	233.04	54.38	4.29	< 0.01	115.57	350.52
	× 1	297.27	46.49	6.40	< 0.01	196.84	397.69

* Statistics analyses data of strain-based regression model in day 4 and force-based regression model in day 4 and 28 are provided in supplemental materials Fig. S3.

**Fig. 6:**
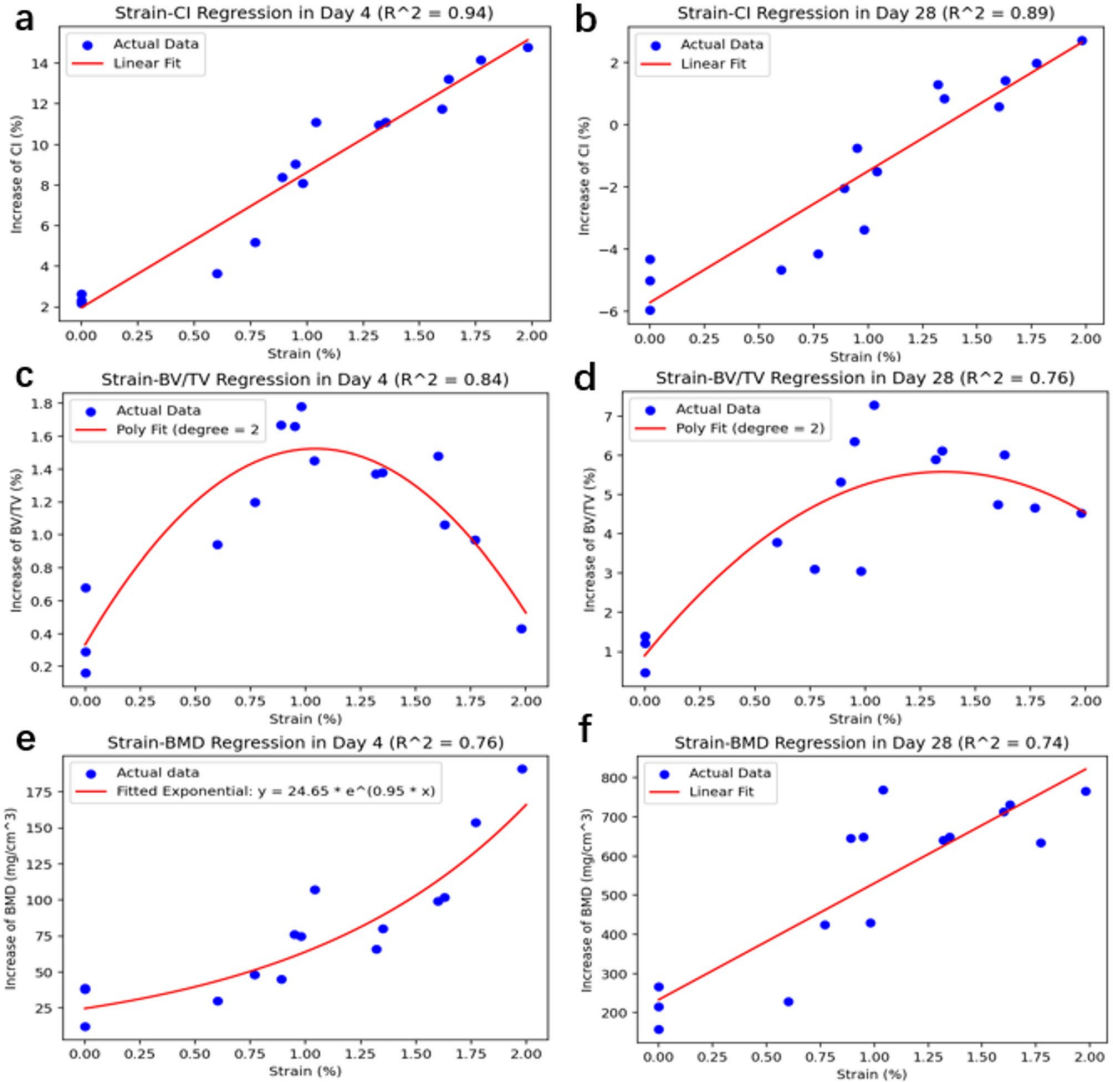
Regression analysis results. (**a**) and (**b**) Relationship between strain and CI in day 4 and day 28. (**c**) and (**d**) Relationship between strain and BV/TV in day 4 and day 28. (**e**) and (**f**) Relationship between strain and BMD in day 4 and day 28.

The strain and CI exhibit a strong linear relationship (R^2^ > 0.9), with high performance in both short-term and long-term prediction, as shown in Fig. 6a and b. As illustrated in Fig. 6c and d, the strain and BV/TV show a quadratic relationship, indicating that the fastest surgical recovery occurs at a strain of approximately 1.0%. However, the regression score for long-term predictions tends to significantly decrease compared with that for short-term predictions. Furthermore, as shown in Fig. 6e and f, the relationship between strain and BMD growth differed at various stages. In the short term, strain and BMD growth follow an exponential relationship, whereas in the long term, a linear relationship prevails.

## Discussion

### Mechanical tension for bone regeneration

Mechanical tension stimulates osteoblast activity to promote bone regeneration; a mechanism widely studied in the literature. Wang et al.^15^ emphasized that the skeletal system’s mechanical properties, mechanosensitive cell populations, and mechanotransduction signaling pathways collectively regulate bone remodeling, with bone formation, resorption, and adaptation relying on mechanical signals. Fu et al.^4^ found in distraction osteogenesis studies that adjusting mechanical loading—such as distraction rate, frequency, or compressive loads—can accelerate bone regeneration. However, mechanical loading has a threshold–both excessive and insufficient tension impair osteogenesis. Checa et al.^16^ demonstrated through mechanobiological models that low-level loading enhances bone formation, while high-level loading inhibits bone formation and capillary growth, offering theoretical insight into spring force thresholds. Borghi et al.^17^ assessed spring-assisted cranioplasty and identified a positive correlation between spring tension and cranial expansion, noting that moderate force effectively expands the skull while minimizing bone damage. Our animal experiments demonstrated that a 50 g spring force was optimal, promoting cranial remodeling while preserving morphology. Bone immunofluorescence staining confirmed superior osteoblast stimulation at this force level, with the greatest fluorescence marker distance observed in the 50 g group. Conversely, forces exceeding 80 g increased the risk of bone damage due to stress concentration.

### Superiority of strain in predicting surgical outcomes

In addition to experimental spring force-based assessment, this study further employed FEA and regression analysis to investigate the relationship between strain and surgical outcomes. Our findings suggest that strain is more effective than spring force in predicting surgical outcomes, providing a stronger foundation for understanding biomechanics. Specifically, we selected the average principal logarithmic strain (also referred to as hydrostatic strain) as the remodeling index due to its suitability for capturing multiaxial deformation and its biological relevance to bone regeneration^18^. While some studies have employed other strain indices—such as Von Mises strain, first principal strain, or tensile strain—these are primarily used to assess yielding or potential failure^19^. In our study, although we did observe certain localized damage in the spring–contact area, our primary focus was on strain-driven stimulation of bone regeneration. Nonetheless, alternative strain indices may be considered in future work to further explore localized damage phenomena.

The relationship between strain and CI was found to be linear, with a high regression score, which aligns with Zhang’s finding on the spring force-CI relationship^20^. These findings suggest that the primary cause of changes in CI is the lateral displacement caused by mechanical loading. For bone regeneration, a quadratic regression between strain and BV/TV indicates a balance between bone regeneration and bone damage^21^. Notably, when strain reaches approximately 1.0%, the increase in the BV/TV reached its peak, which supports Carter’s statement of the optimal mechanical strain for bone regeneration^22^. In long-term observation, the relationship between strain and BMD became linear, which is consistent with the findings of Feng et al. on the linear relationship between small strain and BMD^23^. Additionally, the regression models related to bone regeneration explained approximately 70% of the variance in the dependent variable, leaving 30% unexplained, possibly because some biological factors^24,25^ were not included in the models.

However, the regression analysis revealed several limitations. First, while an exponential relationship between strain and BMD was observed in the short-term response, we hypothesized that the osteogenesis-promoting effect may reach an upper limit, which could become evident with the inclusion of additional data from larger strain values. Second, the long-term regression models were found to be less significant than their short-term counterparts, indicating that the influence of mechanical loading on bone regeneration and cranial deformation diminishes over time. Furthermore, the strain-based models did not show a substantial improvement (with an increase in score of less than 0.1) compared to the spring force-based model. This lack of improvement could be due to insufficient data or the inherent limitations of the FEA model. In future studies, increasing the volume of data and refining the FEA model could enhance the performance and predictive accuracy of the regression models.

### FEA’s predictive capabilities, validation, and limitations

In this study, FEA was employed to predict the stress and strain when SAC treatment was applied. First, it was predicted that mechanical strain exceeding 3.0% resulted in bone crack based on experimental observations, highlighting that this FEA model can identify potential bone damage^26^ before surgery. Second, the simulation results accounted for individual variations in geometry and bone density, enabling more precise predictions of surgical outcomes compared to a standard average model^1^. Third, the FEA model was well supported by imaging analysis and animal experiment, reflecting a more accurate bone elastic modulus and cranial deformation. This study fills this gap because few studies have considered individual differences in and experimental validation of SAC treatment.

In surgical planning, virtual modeling techniques—especially FEA—can predict cranial biomechanical responses to different interventions. For example, Bozkurt et al^27^. developed patient-specific FEA simulations of spring-mediated expansion, allowing surgeons to optimize osteotomy design and spring placement preoperatively. Such computer-aided planning improves reproducibility and has been shown to shorten operative time and minimize complications by tailoring the correction to the patient’s anatomy^28,29^. Such advances align with the current study’s finding that moderate, controlled strain yields the best outcomes. Indeed, excessive distractive forces risk bone damage or relapse, whereas a moderate spring-generated strain (~ 1% in the present analysis) optimally stimulates new bone formation without structural compromise—echoing established mechanobiological principles of gradual cranial expansion. This synergy of FEA-based biomechanical modeling and AI-driven decision support provides a rigorous framework for refining SAC, enabling surgeons to virtually “test” and optimize surgical parameters (spring stiffness, placement, duration) before entering the operating room. By embracing these technologies, future SAC procedures can achieve more predictable and improved outcomes^10^.

However, several limitations should be mentioned regarding the FEA models. It is important to note that this study focused solely on the rat for experimental validation. Even though pre-clinical rat models are widely used for simulating bone healing^30^, directly converting rat model to human model cannot ensure that the accuracy remains high^31^. Furthermore, strain exceeding 1.0% are likely to induce plastic deformation over the bone^14^, however, plasticity was not incorporated into the model due to the lack of relevant material data. The oversimplification of suture assignment may have further contributed to the underestimation of strain^32^, as sutures with a lower elastic modulus are expected to undergo greater deformation under spring loading. These factors may partly account for the discrepancy observed in the experimental results. Moreover, FEA model in this study was limited to short-term responses. Long-term simulations could provide more precise predictions of surgical outcomes, although they would also introduce the complexity of dynamic modeling^33^.

### Innovative integration of experimental and computational approaches

This study innovatively combined animal experiments with computational models to enhance the clinical relevance of SAC treatment for craniosynostosis. By integrating bone immunofluorescence staining, imaging parameters (e.g., BMD, BV/TV), and CI measurements with FEA and machine learning, a multidimensional assessment of disease progression and surgical outcomes was achieved. This approach established quantitative links between strain and bone regeneration, offering valuable insights for personalized surgical strategies. The experimental design was clinically relevant, using 3-week-old rats that developmentally correspond to human infants aged 6–12 months, progressing to an age equivalent to 1.5–2 years by study completion—aligning with typical cranioplasty timing^34^. Postnatal cranial elongation was observed in both species, as evidenced by decreasing CI values during growth. The use of rats in this early postnatal growth stage provides a valuable model for investigating the mechanisms underlying normative and altered cranial development.

However, the use of healthy young rat models in this study may limit the direct applicability of the results to human craniosynostosis due to differences in pathological conditions. Holmes^35^ noted that animal models have limitations in simulating the genetic and developmental complexity of human craniosynostosis, particularly in the context of mutation-related syndromes. Yu et al.^36^ also emphasized that the cranial structure of rodent models differs significantly from that of humans, potentially affecting the clinical translation of the results.

Future research could introduce disease-specific models (e.g., FGFR2 or TWIST1 mutant mouse models) to more accurately simulate human pathological conditions. Additionally, comparative experiments with different spring positions could be conducted to refine spatial mechanical analysis. Furthermore, long-term observation data (> 28 days) could be incorporated to assess the long-term stability of bone remodeling^37–39^. Combining clinical case data to validate the predictive capabilities of the model would also facilitate the translation of research findings into clinical practice. Cross et al.^3^ proposed that patient-specific FEA models combined with high-throughput sequencing technology could optimize surgical planning and predict long-term cranial growth trajectories. Moreover, integrating machine learning algorithms with advanced imaging techniques (e.g., 3D CT and MRI) could further enhance prediction accuracy, supporting personalized treatment^40^. The ultimate goal is to guide surgical design through computational model which maximizes the rate of bone regeneration and control CI within the ideal range, like solving a constrained optimization problem with multiple surgical variables.

## Materials and methods

### Ethics declarations

All animal experiments were approved by the Institute of Laboratory Animal Research, Chinese Academy of Medical Sciences (ILARC), which provided all rats used in this study.

This study was conducted in accordance with the National Research Council’s *Guide for the Care and Use of Laboratory Animals*.

All methods were carried out in accordance with relevant guidelines and regulations and were reported in accordance with the ARRIVE guidelines (https://arriveguidelines.org).

### Animal experiments

*Spring Design*: U-shaped nickel-titanium springs (2 cm total length) were activated at 3 mm compression to generate forces of 30, 50, 80, and 100 g (wire diameters: 0.3, 0.4, 0.5, 0.6 mm). The 0 g group received non-functional springs (Fig. 1).

*Experimental animals*: Fifteen 3-week-old male Sprague–Dawley (SD) rats (weight: 40–60 g) were provided by the Institute of Laboratory Animal Sciences, Chinese Academy of Medical Sciences. All rats were housed in a specific pathogen-free (SPF) animal facility and fed a standard diet. They were randomly divided into five groups (0 g, 30 g, 50 g, 80 g, and 100 g spring groups, n = 3 per group).

*Surgical procedure*: Preoperatively, animals were anesthetized via intraperitoneal injection of a mixture of Zoletil (tiletamine and zolazepam in equal proportions) and xylazine hydrochloride (10:1 ratio) at a dose of 0.1 mL per 10 g body weight. The surgical area was shaved and disinfected, and all procedures were performed under aseptic conditions. A sagittal suture expansion surgery was performed on all rats: a 2-cm sagittal incision was made along the midline of the skull, extending between the orbital ridges and the midpoint between the ears. Scalp tissues were dissected layer by layer to expose the sagittal suture. A rectangular strip of sagittal suture tissue (3 mm in width) was excised using a micro motorized saw, with the anterior and posterior fontanelles serving as the endpoints. A spring was implanted at the midpoint of the excision (Fig. 1b). After ensuring hemostasis, the surgical site was irrigated, and the incision was closed in layers.

*Postoperative monitoring*: Body weight, wound healing, and infection status were recorded. Rats were euthanized via carbon dioxide overdose 28 days post-surgery, and tissues were harvested for analysis.

### Laboratory analysis

*Bone fluorescence labeling*: On days 0 and 14, all rats received intraperitoneal injections of calcein (25 mg/kg body weight; Rhawn, China), and on day 4, tetracycline (25 mg/kg body weight; Rhawn, China) was administered. Prior studies indicate that fluorescent bone labels integrate into newly formed bone during distraction osteogenesis, enabling visualization of sagittal suture remodeling via fluorescence microscopy^41^.

On day 28, after continuous spring traction, all rats were euthanized. Specimens containing the sagittal suture and adjacent cranial tissues were harvested. Soft tissues were carefully dissected, and samples were fixed in 4% paraformaldehyde for 24 h, followed by 70% ethanol for 14 days. Gradual dehydration (70%–100% ethanol) was performed, and specimens were embedded in methyl methacrylate. Coronal Sects. (60 μm thick, ≥ 3 per animal) of the sagittal suture were prepared using a microtome, polished, and analyzed under a fluorescence microscope.

Fluorescence microscopy was employed to acquire scanned images, with calcein exhibiting red fluorescence and tetracycline displaying green fluorescence. According to literature^42^, the fluorochromes typically require 24–48 h to reach and stain the cranial defect area. In the 40 × magnified fluorescence images, parallel lines were drawn at 50 μm intervals along the direction perpendicular to the fluorescence marker lines. At the same horizontal measurement line, the distance between the red fluorescence marker (calcein) and the yellow fluorescence marker (overlap region) was measured (indicated by orange short dashes in the schematic diagram). All measured values were statistically analyzed, and their arithmetic mean was calculated to represent the average spacing between red and yellow fluorescence markers in the sample. Each sample was analyzed based on at least three sections, with results presented in Fig. 2. The sample size was approximately 1 × 1 cm, encompassing the sagittal suture and adjacent cranial regions.

### Imaging analysis

On days 0, 1, 4, 7, 14, and 28, body weight and cranial micro-CT scans were recorded using a Revvity Quantum FX system (USA) with a spatial resolution of 5 μm and exposure time of 3 min. Animals were anesthetized via intraperitoneal injection of a mixture of Zoletil (tiletamine and zolazepam in equal proportions) and xylazine hydrochloride (10:1 ratio) at a dose of 0.1 mL per 10 g body weight during scanning. *Mimics* (Version 25.0, Materialise NV, Belgium; https://www.materialise.com/mimics) reconstructed 3D models to measure cephalic index (CI) and suture defect displacement. Bone mineral density (BMD) and bone volume fraction (BV/TV) were quantified using *Analyze* software (Version 14.0, Biomedical Imaging Resource, Mayo Clinic, USA; https://analyzedirect.com/analyze/).

*To ensure precise quantification of bone volume (BV) and total volume (TV), we implemented the following measures*: Pre-Scan Calibration: The micro-CT system was calibrated using a hydroxyapatite phantom (density gradient: 0–1200 mg HA/cm^3^) to establish a quantitative relationship between grayscale values and equivalent bone mineral density. The calibration curve demonstrated a linear correlation between CT values (y) and the nominal phantom density (x): y = 38.185x + 16,804 (R^2^ = 0.9998). This relationship was subsequently used for threshold determination in bone tissue segmentation.

*Image processing protocol*: Raw scans were reconstructed at the highest resolution (5 µm isotropic voxels) and processed in *Mimics* using a semi-automated segmentation workflow. A fixed global threshold (determined empirically from phantom scans and validated against histology in pilot studies) was applied to differentiate bone (mineralized tissue) from soft tissue. To address partial volume effects at boundaries, we used morphological operations (e.g., dilation/erosion) to refine edges and ensure continuity of segmented structures.

*Validation of segmentation*: Image segmentation was performed using a grayscale threshold-based semi-automated method for initial screening, followed by extensive manual refinement. The validation of segmentation was rigorously conducted for each sample by overlaying the generated 3D model (from Mimics) onto the original grayscale slices in two orthogonal planes (sagittal and coronal). All discrepancies identified during this validation process were manually corrected by two independent operators ([Cheng Wenjie] and [Hou Bozhi]), who processed all sample CT sequences in a blinded manner. This manual correction focused specifically on two critical regions: (1) the precise elimination of small sinus cavities/voids in the anterior cranial vault to ensure these non-bone regions were excluded from the bone volume (BV), and (2) the careful delineation of the blurry boundaries between the calvarial bone and adjacent soft tissues, which was verified layer-by-layer to ensure contour accuracy. Throughout the entire procedure, a consistent set of predefined anatomical landmarks was used to guarantee standardized processing across all samples.

*Cranial length (Long Axis)*: Maximum distance from the nasal point (intersection of nasal and frontal bones in the midsagittal plane) to the occipital point (superior margin of the foramen magnum).

*Cranial width (Short Axis)*: Maximum lateral distance across the skull.

*Region of interest (ROI)*: A plane parallel to the short axis or encompassing both long and short axes was defined in 3D reconstructions. The cranial region above this plane was designated as the ROI (Fig. 3).

*Cephalic index (CI)*: Cranial Width/Cranial Length × 100.

*Bone mineral density (BMD)*: Quantified within the ROI.

*Bone volume fraction (BV/TV)*: Ratio of bone volume to total cranial volume in the ROI, expressed as a percentage.

### Finite element analysis

*Model construction*: FEA was performed to simulate the biomechanics of SAC treatment in rats using *ABAQUS* (version 6.14, Dassault Systèmes Simulia Corp., USA; https://www.3ds.com/products-services/simulia/products/abaqus/). For each rat, finite element models were developed from reconstructed 3D geometry to account for variations in cranial shape, bone density, and spring force.

*Meshing*: Considering that the thickness of the rat craniofacial region is approximately 0.5 mm in the animal tests, an adaptive mesh with a maximum size of 0.5 mm was used, employing C3D10 elements to represent the cranial geometry. As part of the adaptive strategy, mesh refinement is automatically applied in regions with high geometric complexity or stress gradients. In particular, the region where the calvarial thickness is relatively low triggered finer meshing, with local element sizes reaching approximately 0.2 mm.

*Section Assignment*: Geometrical section included cortical bone and the sutures, similar to the relevant research of human infant^3^. The location and width of sagittal suture, coronal suture and lambdoid suture were obtained from the reconstructed 3D model. In our finite element model, the cranial sutures were represented using simplified geometries without fully capturing the complex interdigitated or overlapping structures visible in high-resolution micro-CT. The remaining part is considered as cortical bone, since the cortical bone essentially shares majority of stress due to stress shielding effect^43^. The section assignment was presented in the supplemental material Fig. S1.

*Material properties*: The cortical bone’s Young’s modulus was calculated individually for each rat based on the subject-specific bone mineral density (BMD) extracted from the micro-CT data, by the equation $E=8362.8({\rho )}^{2.56}$. The equation follows the empirical relationship: where $E$ is the elastic modulus in MPa, $\rho$ is the BMD in g/cm^3^, and the constant values are fitting parameters^13^. This ensures that mechanical properties reflect inter-subject variability. All bones were therefore assigned non-uniform values depending on their corresponding density distributions.

The Young’s modulus of the sutures was 4.72 MPa, refer to Henderson et al^44^. A Poisson’s ratio of 0.3 was applied uniformly across all models. All the sutures were assigned the same value for each rat model.

*Boundary and loading conditions*: Boundary conditions were defined to be fixed in 6 degrees of freedom and assigned to the boundary of ROI. A concentrated force was applied to simulate the placement of the springs.

*Analysis type*: A quasi-static analysis was conducted to model short-term biomechanical behavior, such as the initial stress and strain responses. Long-term effects were not considered in this study due to the complexity of incorporating the factors such as natural growth.

*Convergence study*: A mesh convergence analysis was conducted for rat A using uniform mesh sizes of 0.1, 0.2, 0.3, and 0.5 mm. The maximum von Mises stress was used as the criterion to assess convergence. The results demonstrated that the difference in maximum von Mises stress between the 0.5 mm and 0.1 mm mesh sizes was within 5% (specifically, 4.9%), indicating sufficient numerical accuracy while maintaining lower computational cost. The detailed mesh convergence study is provided in the supplementary materials Fig. S2.

*Model validation*: The accuracy of the FEA model was assessed by comparing simulated and experimental changes in wound width following spring implantation. Lateral expansion of the long edge of the cranial defect was quantified in both the FEA simulations and experimental rats at two time points: immediately before spring implantation (day 0) and one day post-implantation (day 1).

Each long edge was divided into ten equal segments, yielding nine evenly spaced internal points, with Point 5 corresponding to the midpoint. At each point, postoperative displacement was defined as the change in inter-edge distance between day 0 (*a*) and day 1 (*b*), i.e., displacement = (*b*–*a*), representing the degree of defect expansion.

In the FEA, displacements at all nodes along the edge were readily obtained, and a displacement curve was fitted using the nine points. For the experimental images, Points 3–7 were selected to correspond to the relevant segment of the FEA-derived curve. Considering simplification in the setup, the FEA model was deemed reliable if the deviation between experimental and simulated displacements was within 10%. The results of model validation were shown in Fig. 5d.

### Regression analysis

*Regression model construction*: Regression analysis was implemented using Python 3.10, the Scikit-learn package (version 1.6.0) and the Statsmodels package (version 0.14.4).

*Regression types*: The relationship between biomechanics and bone regeneration was unclear, but we hypothesized that it would not exhibit a high nonlinearity. Hence, linear regression, polynomial (quadratic) regression, and exponential regression were tried to model the relationships between biomechanical indicators and surgical outcomes.

*Biomechanics datasets*: Mechanical strain is believed to be the primary factor driving bone remodeling^45^. This study selected average principal logarithmic strain as the index based on relevant research on bone remodeling in computational biomechanics^46^. Additionally, we consider spring force as a comparison index.

- Average principal logarithmic strain of ROI (hereafter referred to as ‘strain’)
- Spring force

*Surgical outcomes datasets*: We used surgical data in day 4 to represents short-term effect, while data in day 28 represents long-term effect. Hence, the dependent variables used to represent surgical outcomes were:

- CI increase on Day 4 and Day 28
- BV/TV increase on Day 4 and Day 28
- BMD increase on Day 4 and Day 28

*Acceptance criteria*: A regression model with an R^2^ score greater than 0.7 was considered acceptable for predicting the relationships between biomechanics and surgical outcomes. If R^2^ score of the quadratic regression model is greater than that of the linear regression model by more than 0.05, then select the quadratic regression model; otherwise, use the linear regression model. This approach is to prevent overfitting.

### Statistical analysis

GraphPad Prism 9.0 was used for statistical analysis. Data are presented as mean ± SD. Permutation tests were applied for animal experiment data, while one-way ANOVA and t-tests assessed significance for other datasets (*p* < 0.05).

## Conclusion

This study elucidates the critical interplay between stress distribution and osteogenesis in spring-assisted cranioplasty. Moderate strain stimulates bone regeneration, while excessive force risks structural compromise. The linear strain-CI relationship enables precise CI control via spring force modulation. By merging FEA and regression analysis, the proposed predictive framework advances personalized cranial surgery design, offering a scientifically grounded paradigm for clinical parameter optimization. These findings establish a foundation for future translational research in craniofacial reconstruction.

## Supplementary Information

Below is the link to the electronic supplementary material.

Supplementary Material 1

## Data Availability

The data are provided within the manuscript or supplementary information files. Source code for the regression and finite element models is available from the corresponding author upon reasonable request.
