# Supplementary material for: Predicting surgical outcomes in spring assisted cranioplasty via finite element analysis and animal experiments
Source: Sci Rep. 2025 Dec 3;15:43058. doi: 10.1038/s41598-025-27092-9 (PMC12675514; doi:10.1038/s41598-025-27092-9)
Supplement: Supplementary file 1 — Supplementary Material 1 [file 41598_2025_27092_MOESM1_ESM.pdf]

Fig S1. Finite element modelling – Section assignment and boundary condition

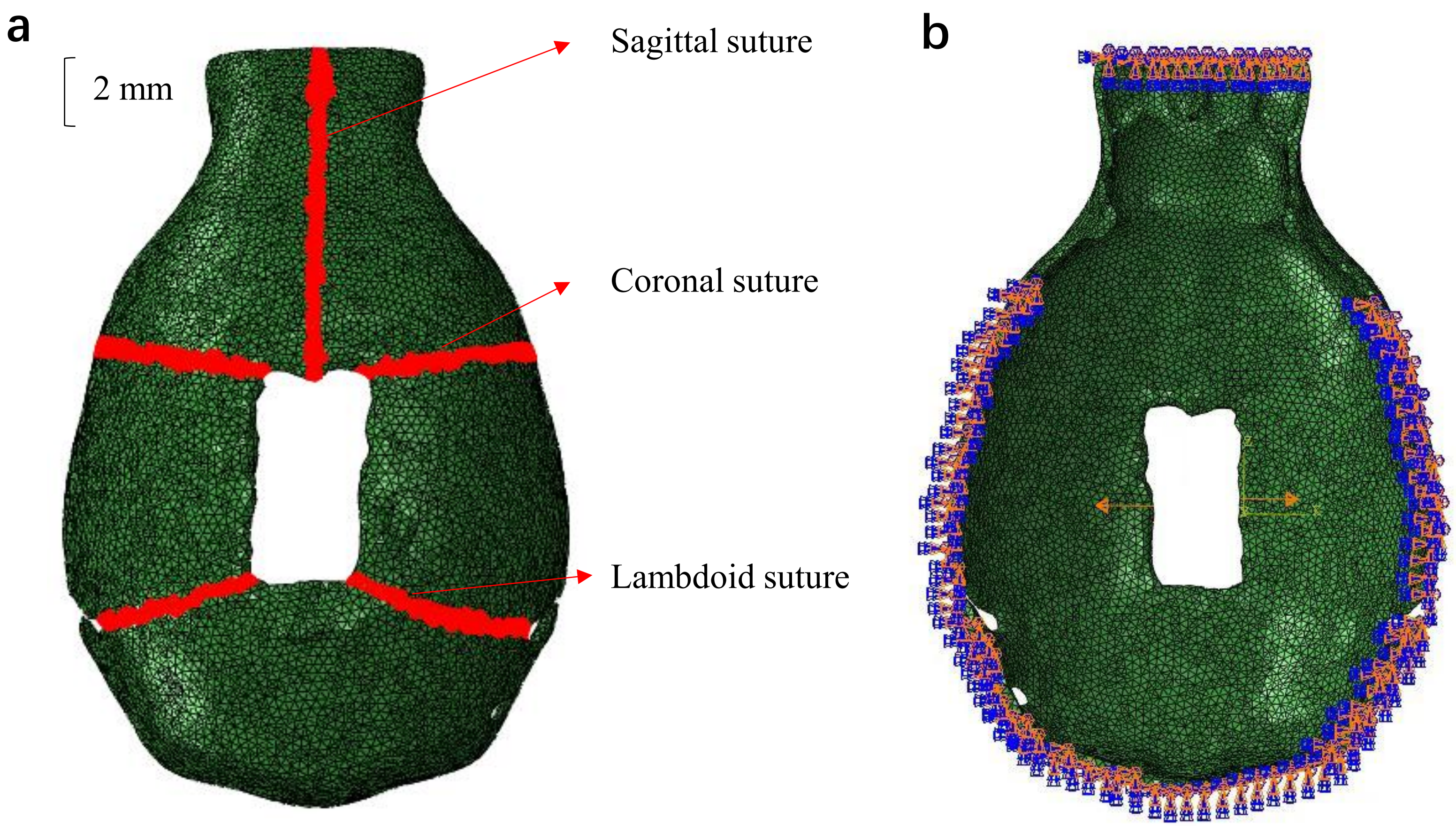

Fig S2. Finite element modelling – Mesh convergence analysis

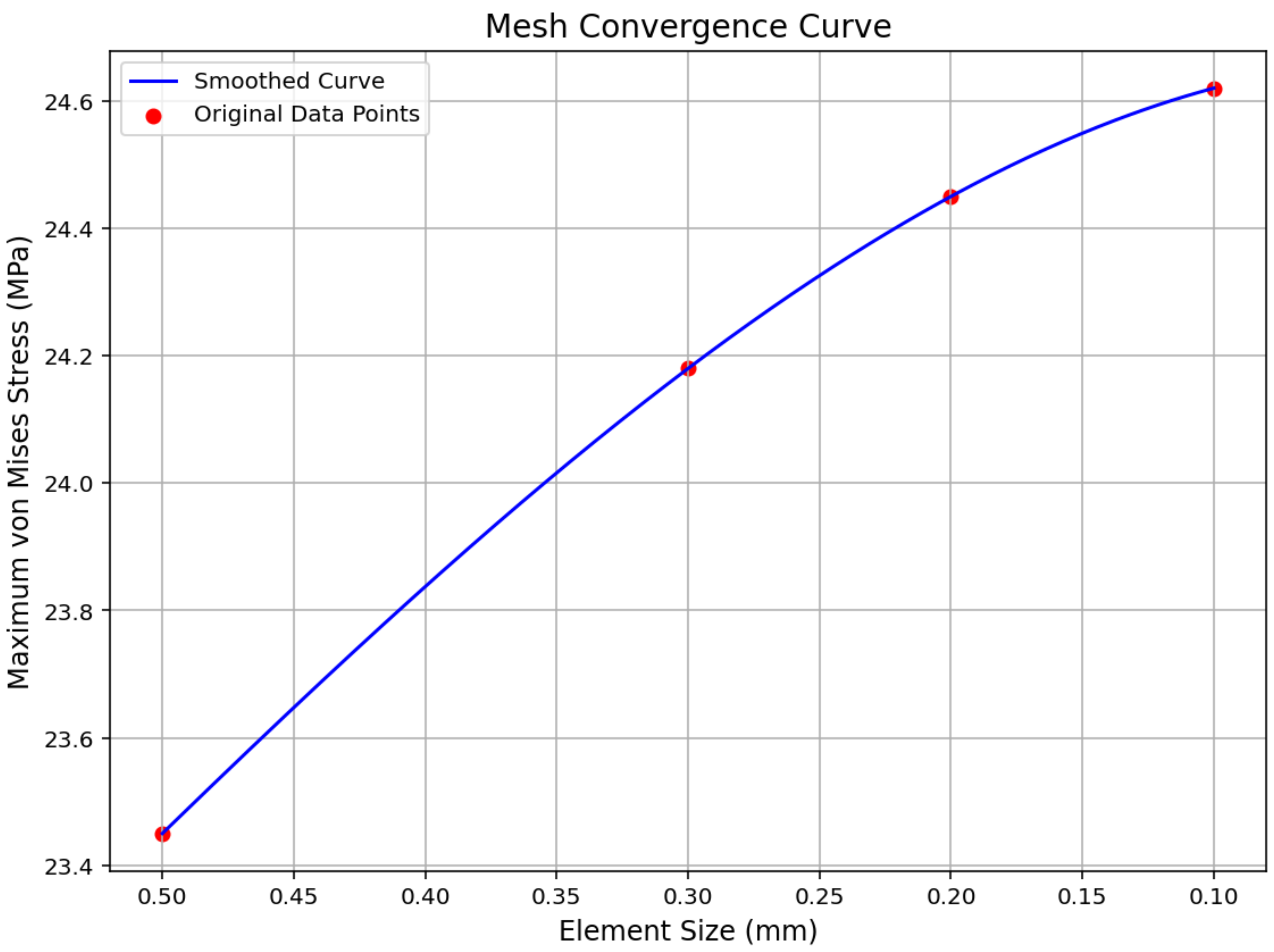

Fig S3. Regression analysis – statistically analyses of regression models

Strain-Based Regression Model in Day 4

		Coeff.	Std err	t	P>|t|	[0.025	0.975]
CI	const	-5.72	0.52	-11.01	<0.01	-6.84	-4.60
	x1	4.22	0.44	9.51	<0.01	3.26	5.18
BV/TV	const	0.33	0.13	2.65	0.021	0.06	0.61
	x1	2.28	0.29	7.96	<0.01	1.66	2.91
	x2	-1.09	0.15	-7.29	<0.01	-1.42	-0.77
BMD	const	18.48	22.45	0.82	0.425	-30.02	66.99
	x1	63.36	19.19	3.30	<0.01	21.89	104.82

Force-Based Regression Model in Day 4

		Coeff.	Std err	t	P>|t|	[0.025	0.975]
CI	const	-5.46	0.40	-13.73	<0.01	-6.32	-4.60
	x1	0.075	0.006	11.95	<0.01	0.062	0.089
BV/TV	const	0.39	0.18	2.21	0.047	0.006	0.780
	x1	0.04	0.008	4.94	<0.01	0.023	0.059
	x2	-4e-4	7.8e-5	-4.54	<0.01	-0..001	0.0
BMD	Regression score not reach the criteria						

Force-Based Regression Model in Day 28

		Coeff.	Std err	t	P>|t|	[0.025	0.975]
CI	const	2.66	0.676	3.93	<0.01	1.12	4.12
	x1	0.11	0.011	10.59	<0.01	0.091	0.137
BV/TV	const	0.72	0.452	1.59	0.137	-0.265	1.706
	x1	0.15	0.021	7.33	<0.01	0.109	0.199
	x2	-0.0014	0.0	-5.64	<0.01	-0.002	-0.001
BMD	const	187.01	55.76	3.35	<0.01	65.53	308.49
	x1	10.90	2.59	4,22	<0.01	5.27	16.53
	x2	-0,057	0.025	-2.32	0.039	-0.111	-0.004
